# 2D perovskite-based high spatial resolution X-ray detectors

**DOI:** 10.1038/s41598-021-02378-w

**Published:** 2021-11-24

**Authors:** Amlan Datta, John Fiala, Shariar Motakef

**Affiliations:** CapeSym, Inc., 6 Huron Drive, Natick, MA 01760 USA

**Keywords:** Materials for devices, Electronics, photonics and device physics

## Abstract

X-ray radiography is the most widely used imaging technique with applications encompassing medical and industrial imaging, homeland security, and materials research. Although a significant amount of research and development has gone into improving the spatial resolution of the current state-of-the-art indirect X-ray detectors, it is still limited by the detector thickness and microcolumnar structure quality. This paper demonstrates high spatial resolution X-ray imaging with solution-processable two-dimensional hybrid perovskite single-crystal scintillators grown inside microcapillary channels as small as 20 µm. These highly scalable non-hygroscopic detectors demonstrate excellent spatial resolution similar to the direct X-ray detectors. X-ray imaging results of a camera constructed using this scintillator show Modulation Transfer Function values significantly better than the current state-of-the-art X-ray detectors. These structured detectors open up a new era of low-cost large-area ultrahigh spatial resolution high frame rate X-ray imaging with numerous applications.

## Introduction

X-ray imaging is the most common and widely used diagnostic technique that spans numerous fields [^1^ and references within]. X-rays interact with the atomic electrons resulting in higher absorption cross-sections for higher atomic number elements depending on the overall electron density distribution of the object. X-ray radiography is currently performed primarily using direct and indirect techniques, which involve the detection of charge carriers and photons generated by the X-rays, respectively. Indirect flat panel X-ray imagers (FPXIs) with scintillating layers (such as commercially available microcolumnar CsI and Gd_2_O_2_S) have high detective quantum efficiency (DQE) and are the preferred detectors for all hard X-ray imaging applications. However, to limit the spreading of the scintillation light, the thicknesses of these sensors are limited to about ~500µm. These sensors provide decent spatial resolutions with modulation transfer function (MTF) values around 30% at 2 lp/mm^1^, but the lower thickness limits the detector sensitivity, resulting in higher X-ray dose requirements. Direct detectors are good candidates for achieving higher spatial resolution. However, charge trapping and defect-related challenges lower the sensitivity of these detectors significantly^2–4^. Due to their lower atomic numbers, the most successful large area direct detectors such as  amorphous selenium (a-Se) and silicon have low efficiencies for higher X-ray energies.

Spatial resolution is an extremely important factor for X-ray imaging that helps users distinguish between two adjacent features. A detector with high MTF, for example, can reliably detect a micron-scale cancerous lesion or an mm-scale fracture in a gas pipeline with high levels of confidence. While the trend of the imaging industry is shifting towards feature recognition using artificial intelligence, higher spatial resolution in radiography images significantly enhances the detection probabilities of subtle features such as pulmonary nodules using the neural network deep learning models^5^. In addition to spatial resolution, the indirect detectors must provide high enough contrast, be manufacturable in large areas (> 10 cm × 10 cm), have a fast decay time, and have a low afterglow. This paper demonstrates a detector that provides an excellent solution for high spatial resolution X-ray radiography. The detector consists of a solution-processable two-dimensional (2D) hybrid perovskite single-crystal scintillator, lithium-alloyed phenethylammonium lead bromide (PEALPB) incorporated into a glass microcapillary array. In terms of crystal structure, hybrid (containing organic and inorganic constituents) perovskite scintillators can be classified as three-dimensional (3D) and 2D. Although the 3D perovskite scintillators with lower exciton binding energy (tens of meV) have been shown to provide excellent X-ray scintillator response^6,7^, the 2D perovskite scintillators have the potential for providing higher light yield and faster decay due to their higher exciton binding energy (hundreds of meV)^8,9^. The 3D lead halide perovskites can be transformed into a 2D one by introducing a long alkyl chain or a bulky organic cation. PEALPB belongs to the 2D family, where the alternating inorganic/organic layers effectively confine the exciton inside the inorganic layer generating scintillation in response to X-rays, fast neutrons, and alpha particles that are released as a byproduct of thermal neutron absorption by Li. Li-doping provides a unique benefit for perovskite materials. Even though the solution-processed hybrid perovskites have demonstrated excellent performance in many applications, a high concentration of trap states with a density of 10^15^–10^16^ cm^−3^ still exists, resulting in nonradiative recombination. It has been experimentally verified that this deleterious feature can be significantly reduced when the lattice is doped with high concentrations of Li^10^. It is possible to synthesize the 2D scintillators with high concentrations of ^6^Li due to the small size of the Li-ion relative to the large unit cell of 2D materials. Li-alloying also broadens the radioluminescence emission spectra of PEALPB, with its maximum at 436 nm^11^. Li-doping also substantially increases the light yield of the PEALPB scintillators^11,12^. In our study, the light yield of free-standing single-crystal PEALPB sensors was found to be around 18,700 ± 1200 ph/MeV as measured using ^137^Cs gamma source. Although the possibility of a microcapillary-based PEALPB sensor fabrication was shown in an earlier publication, very limited success in X-ray imaging was achieved due to the unoptimized sensor fabrication^12^. The current study has successfully demonstrated the extreme spatial resolution limits of X-ray imaging using this approach. In addition, the presence of ^6^Li provides a large capture cross-section for the detection of thermal neutrons, while the high concentration of hydrogen (24 hydrogen atoms/molecule) enables the detection of fast neutrons. Thus, by adjusting the content of ^6^Li in the matrix, these detectors can also be tuned to detect neutrons over a wide range of energies. The results showing the neutron detection capabilities are provided in the Supplementary part of this paper.

We will demonstrate in this paper that the spatial resolution of these detectors with thicknesses as high as 1200 µm was found to be better or comparable to the state-of-the-art direct and indirect detectors with much lower thicknesses. The decay time constant of the PEALPB 2D perovskite proposed here has been measured to be about 11–24 ns, and its afterglow is an order of magnitude lower than the modern industry-standard CsI scintillators^13^. Thus, compared to the CsI scintillator-based detectors, the maximum count rate of detectors based on PEALPB can be expected to be appreciably higher. Although no specific radiation hardness studies have been done for PEALPB, in general, the radiation tolerance of the 2D perovskites has been shown to be very high^14^, which makes them appropriate for high flux X-ray synchrotron beamline and industrial applications.

## Results and discussion

**Figure 1:**
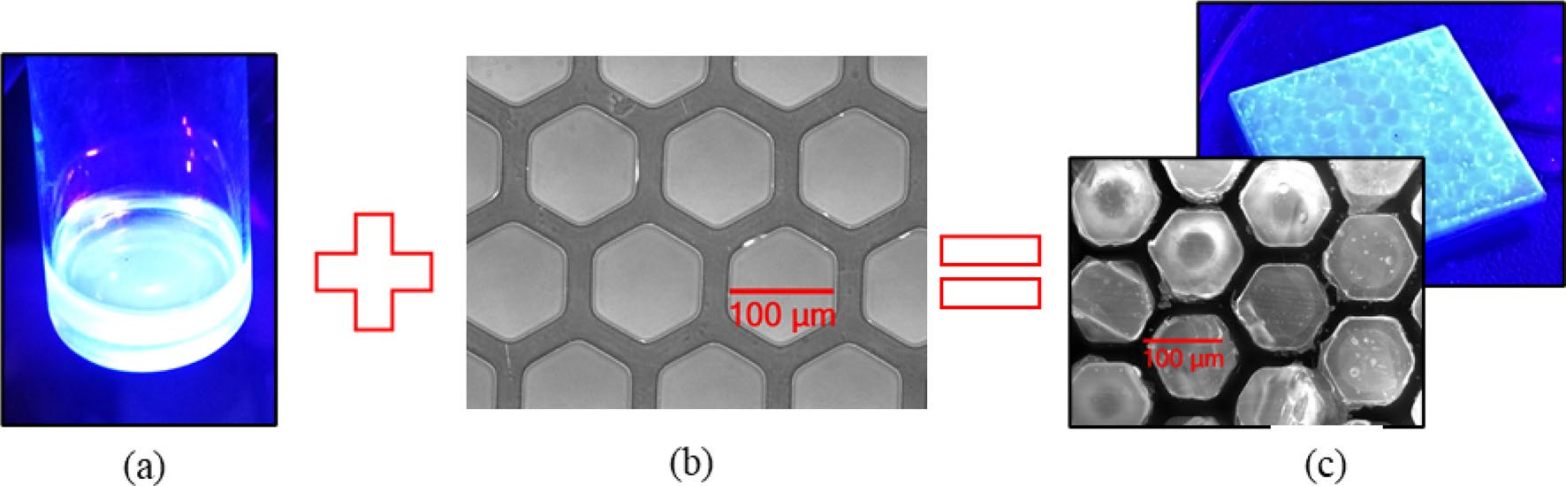
The production of the dual imaging detector using PEALPB produced by wet chemistry (**a**) and microcapillary plates (**b**) to produce a high-resolution imaging detector (**c**) with single-crystalline PEALPB scintillators.

Achieving high detection efficiency is necessary to enable high-energy X-ray imaging. For all known scintillators, this can be achieved by the use of thick films of the order of several millimeters. For example, for 50% attenuation of 150 keV X-rays, CsI needs a thickness of 2.1 mm. Such film thicknesses, however, are not conducive to high spatial resolution imaging, as the photons generated in the film propagate isotropically and impinge on a multitude of pixels on the imaging chip resulting in blurred images. To overcome this issue, we focused on using microcapillary plates with pore sizes between 20 and 100 µm. These plates are available in a variety of thicknesses (from sub- to several-mm) and cross-sectional areas (up to 500 cm^2^). We successfully filled 100 and 20-micron pore diameter microcapillary plates with single-crystalline PEALPB perovskite scintillator with similar X-ray attenuation coefficients compared to CsI (e.g. 1.9 vs. 1.8 cm^2^/g at 100 keV) and demonstrated an X-ray imager using this sensor. Figure 1 summarizes the overall approach to the production of the microcapillary-based detectors. Figure 1c shows a magnified fluorescence view of the microcapillaries containing the single crystals of PEALPB. The back image in Fig. 1c shows a 2 cm × 2 cm × 1.2 mm microcapillary plate scintillating under UV irradiation. The arriving X-rays from the source ionize the scintillating material in the microcapillary, producing photons that emanate isotropically. The index of refraction of PEALPB is appreciably higher (~ 1.9–2.1) than that of the capillary walls made from silica (~ 1.5). The refractive index of the PEALPB scintillators was measured using a refractive index meter. The photons undergo nearly complete internal reflection along the glass capillary before they leave the detector and enter the photon collecting photodetectors.

**Figure 2:**
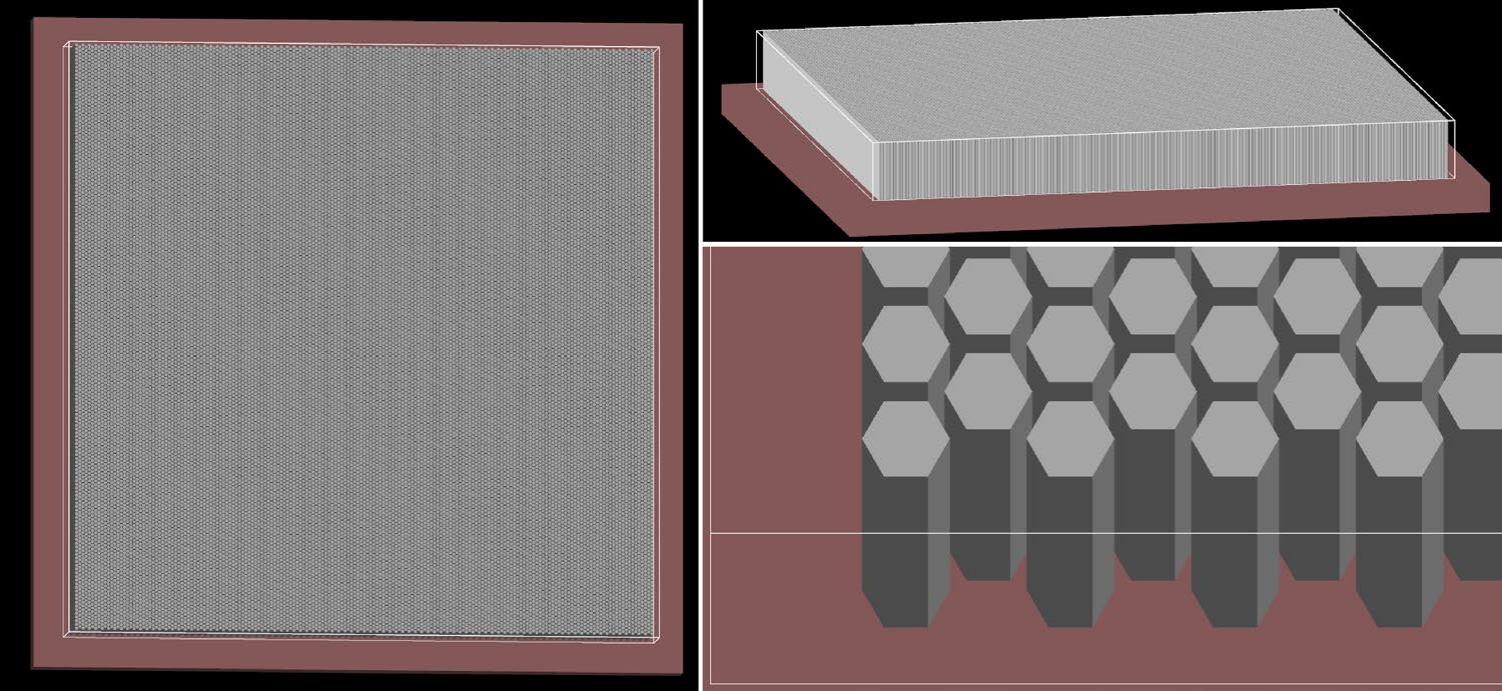
Geant4 model of borosilicate glass faceplate with 100 µm hexagonal capillaries filled with PEALPB similar to the microcapillary plates shown in Fig. 1. Left image: top view of 2 × 2 cm faceplate on top of silicon imager. Upper right: Side view of the model. Lower right: Higher magnification of the 1.2 mm tall PEALPB scintillators in a glass matrix.

**Figure 3:**
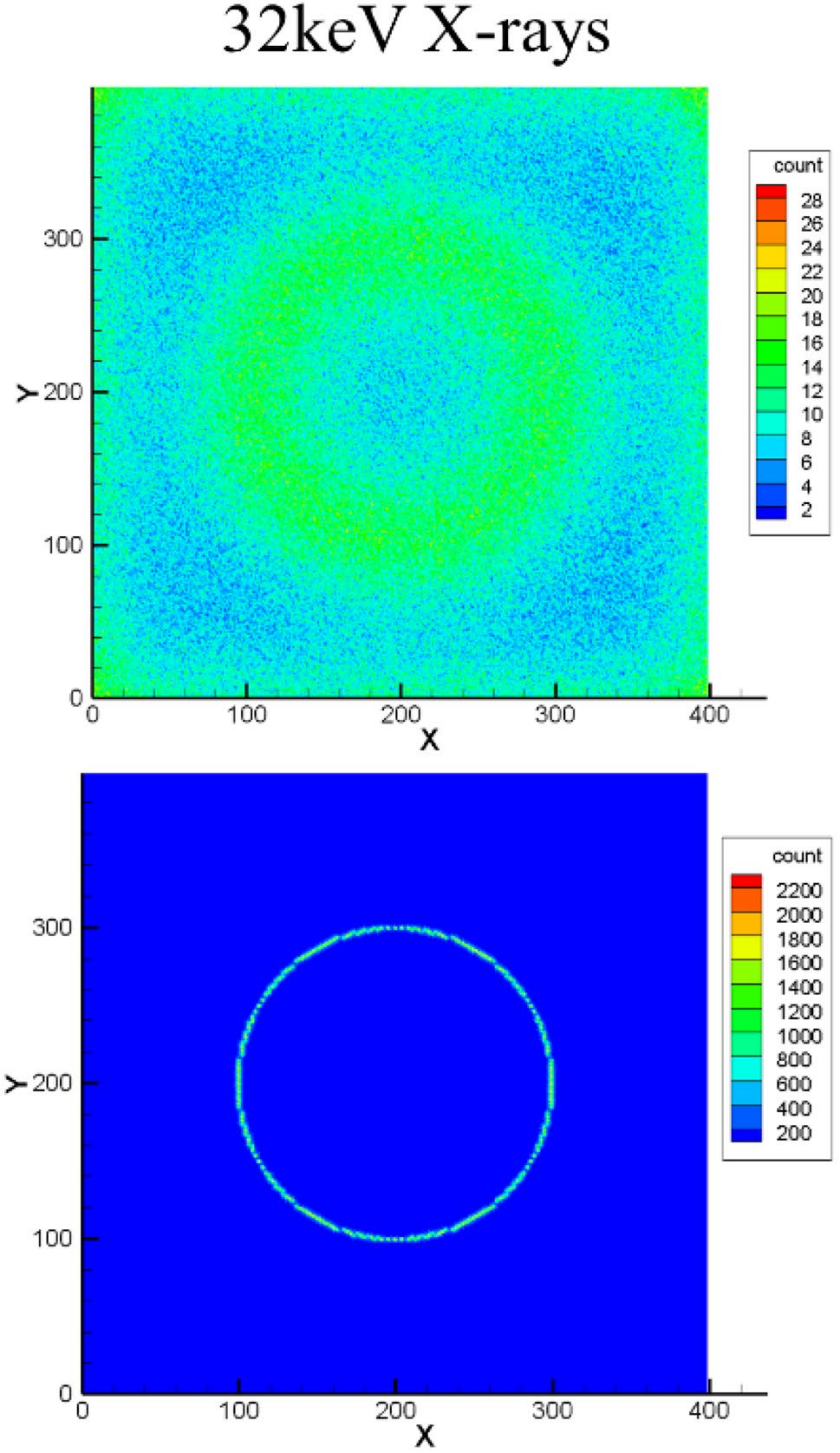
Hit counts of optical photons on pixels of the silicon imager generated using a circular pattern of incident 100,000 hard X-rays (32 keV) on the array of PEALPB detectors. Top row hit map shows the light generation without a microcapillary plate and the bottom row shows the same with the microcapillary plate.

Geant4 simulations were used to assess the effectiveness of microcapillaries to achieve a high spatial resolution. A Geant4 model of a high-density array of 29,000 hexagonal PEALPB detectors was constructed (Fig. 2). The faceplate was placed on a silicon imager consisting of 400 × 400 square pixels of 50 µm on a side. A circular pattern of radiation, either 32 keV hard X-rays (and 25 meV thermal neutrons) was directed at the faceplate from above to assess the ability of the design to provide good imaging resolution. Energy deposited by the radiation in PEALPB generated optical photons that scattered throughout the faceplate. However, as shown in Fig. 3, the difference in refractive index between PEALPB and glass tended to confine the photons to the hexagonal capillary, resulting in most photons being collected directly beneath the point of interaction. These simulations verify our hypothesis of obtaining very high spatial resolution detectors using this detector design. The thermal neutron results are shown in Fig. S3. We also performed Geant4 modeling for the fast neutron response of the PEALPB detectors, where we obtained comparable fast neutron responses of these detectors to the state-of-the-art Stilbene (supplementary section S1).

**Figure 4:**
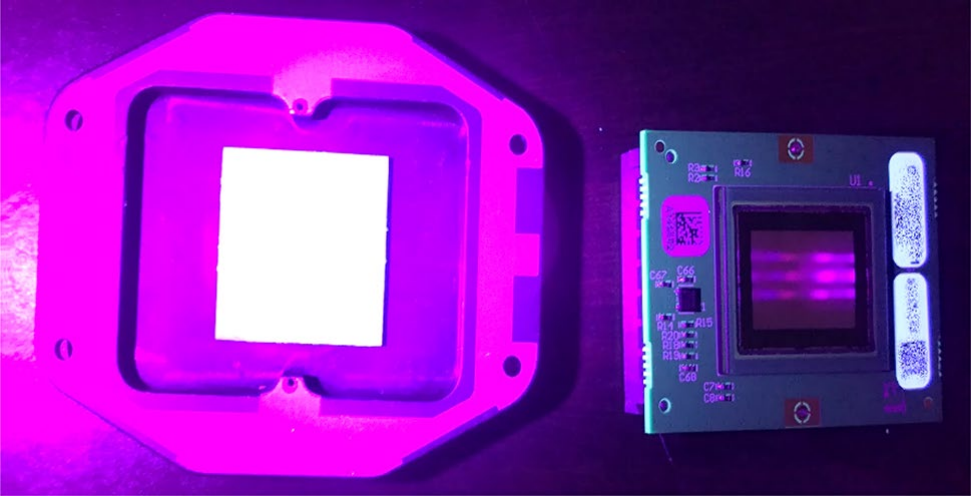
(Left) PEALPB detector attached to a metal plate that couples with the (right) CMOS sensor.

The following section will demonstrate the X-ray imaging characteristics of the PEALPB-based detectors. The neutron detection characterization setup and results are provided in the supplementary section S1. The neutron detection performance of these detectors in terms of neutron sensitivity exceeded the commercial sensors used for neutron radiography (Figs. S5 and S6). For X-ray imaging, a camera was constructed using the 2 cm × 2 cm microcapillary plates where the microcapillaries are filled with single crystals of PEALPB, as shown in Fig. 1. A microcapillary diameter of 20 µm and a CMOS chip pixel pitch of 3.75 µm was used to construct this high spatial resolution indirect camera. Figure 4 shows the PEALPB detector and the CMOS chip that was used to construct the camera under UV irradiation. This camera was placed in line with a microfocus X-ray source, and the imaging objects were placed on an XYZ stage in between.

**Figure 5:**
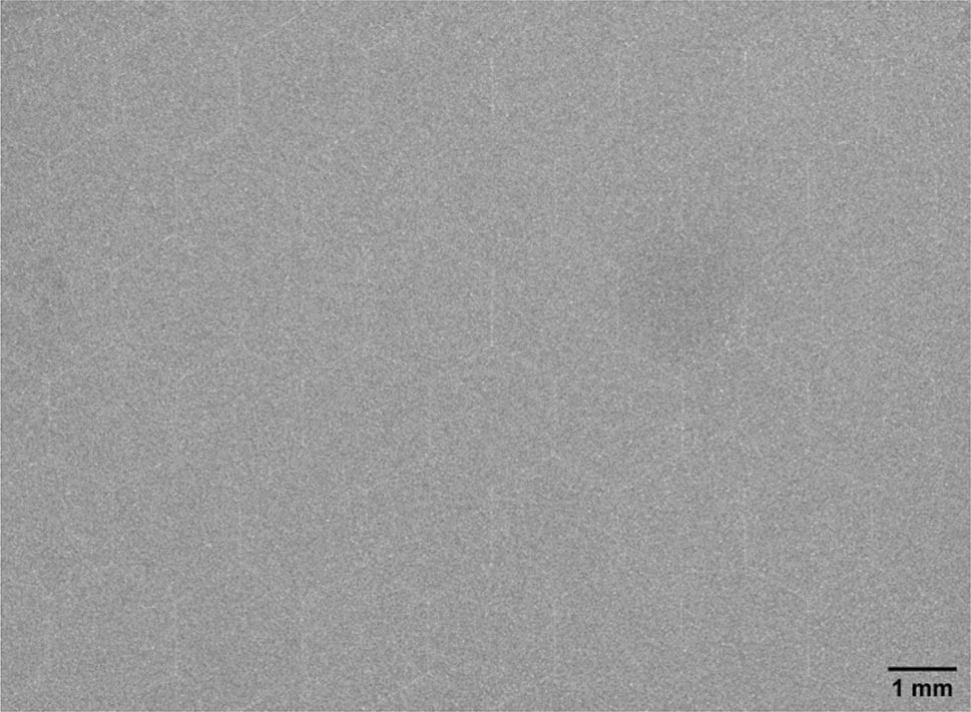
Background X-ray image of the PEALPB camera.

**Figure 6:**
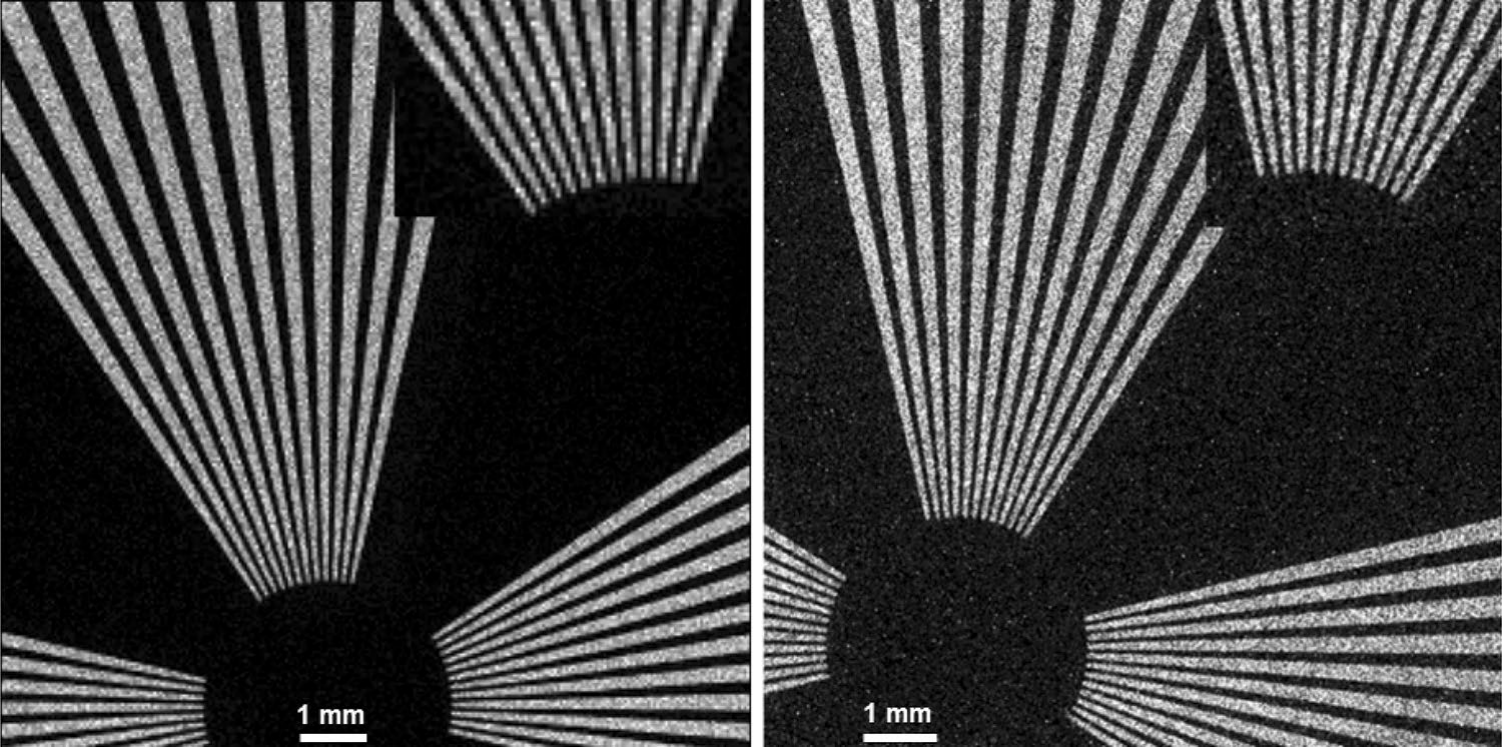
X-ray resolution target images using (left) Timepix camera with a Si sensor, and (right) PEALPB camera. The insets in the picture show the magnified area of the highest resolution end of the resolution target. The innermost lines have a Nyquist frequency of 10 l p/mm.

**Figure 7:**
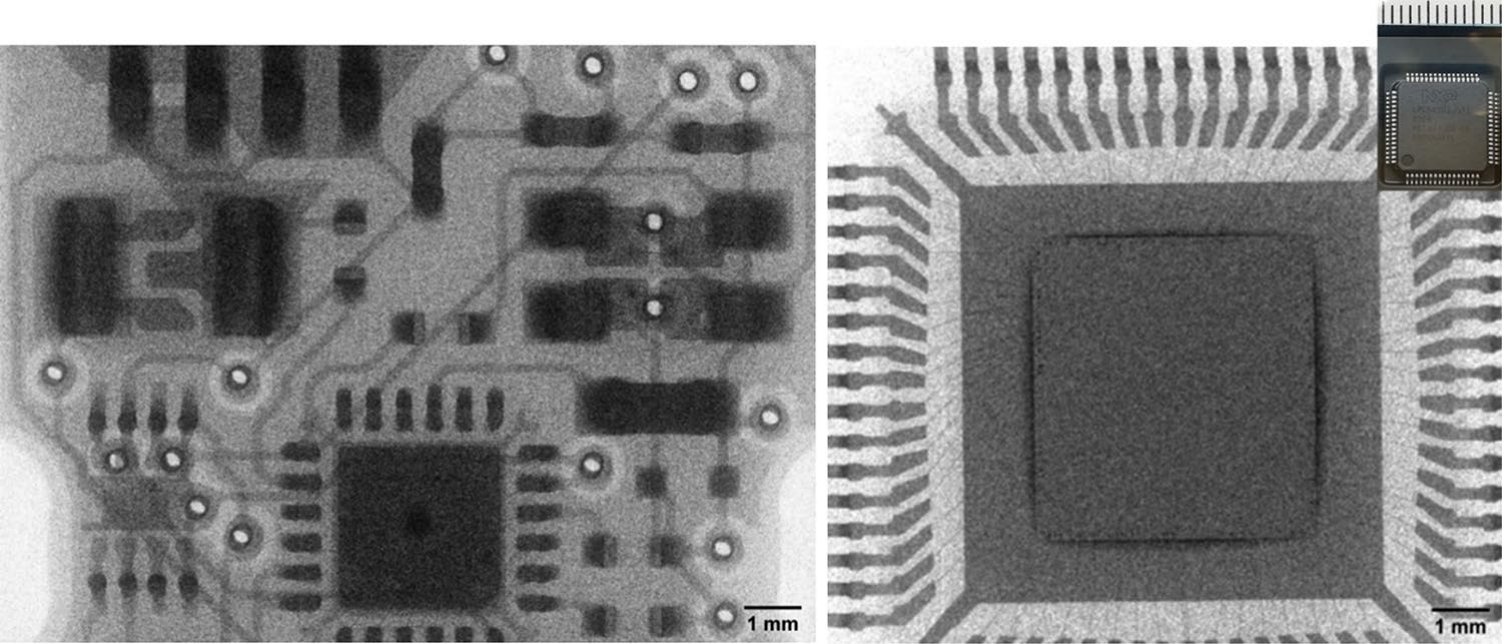
X-ray images of an USB-drive circuit and a microchip. The inset shows the physical mm-scale microchip.

First, the uniformity of the background was measured without any imaging object between the camera and the X-ray source. The background image acquired using 90 kV X-ray is shown in Fig. 5. Seven regions of interests (ROIs) with 200 × 200 pixels were used to perform the uniformity analysis. The uniformity of the background image was found within 0.056% with about 4% noise. The detailed ROI stats for these regions are provided in Table S1. Figure 6 shows the image of an X-ray resolution target taken using the PEALPB camera and a Timepix camera with a silicon sensor. The excellent spatial resolution of the PEALPB camera is evident. The relative contrast measured on the raw image for PEALPB at the lowest frequency of the X-ray resolution target gives an average of 491.7 compared to 531.8 for the Si detector. The signal-to-noise ratio (SNR) for this area is measured to be 78. Figure 7 shows the X-ray image of a mini USB drive circuit. The tiniest features are well resolved.

**Figure 8:**
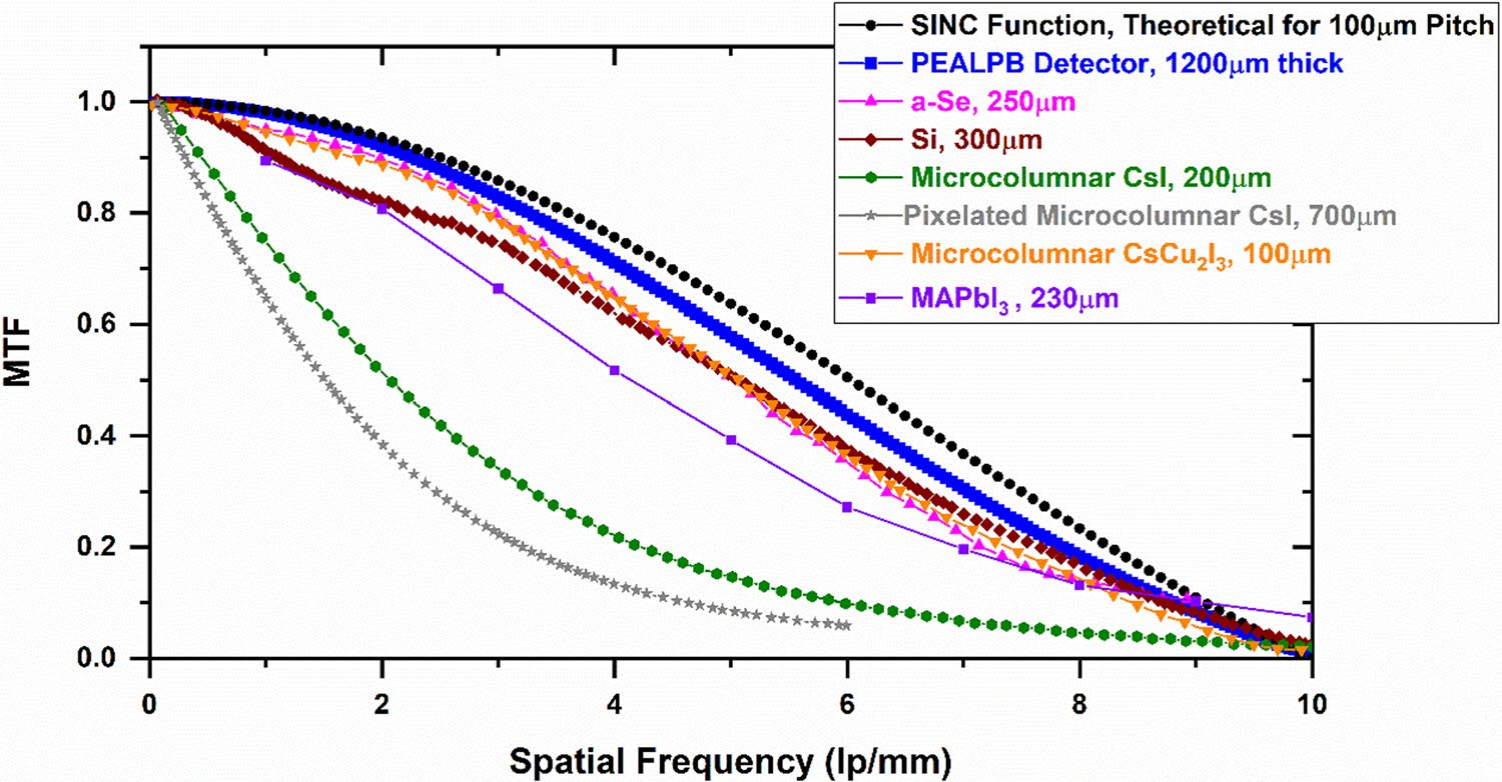
MTF curve of PEALPB detectors along with other state-of-the-art detectors with relatively lower thicknesses. The plot thicknesses are representative of the sensor thickness levels. The sync function plot corresponding to a 100 µm pixel pitch is also shown for reference.

The MTF of this detector was calculated using the slanted edge technique. Figure 8 shows the MTF versus spatial frequency plot derived from the PEALPB camera. This figure also summarizes the MTF values for different other direct and indirect sensors. This comparison has not been done under the same experimental conditions (such as X-ray dose or energy). However, as these are the best-achieved values for these sensor materials, Fig. 8 clearly shows the enhanced spatial resolution of the microcapillary-based PEALPB detectors over the other state-of-the-art sensors. For the best indirect sensor materials such as microcolumnar CsI or the newly discovered CsCu_2_I_3_ with microcolumnar structures, the MTF values decrease sharply with film thicknesses [Fig. 2 of ref^15^, Fig. 3 of ref^16^]. In addition, for semiconductor detectors increasing thickness enhances charge trapping and detector noise resulting in reduced detector sensitivity and bandwidth. However, with the microcapillary-based detector structure, we demonstrated that even with thicknesses as high as 1.2 mm, the detector spatial resolution, as well as its sensitivity to X-rays, can be simultaneously well maintained. The best available spatial frequencies with 10% and 50% MTF for different detectors are summarized in Table 1. Higher sensor thicknesses are necessary to increase the efficiency and sensitivity of the detectors. None of the existing technologies can provide a solution for fabricating detectors with higher thicknesses without critically affecting the spatial resolution. The data in Fig. 8 also shows that the multimodal X&N detector based on PEALPB can provide high spatial resolution matching an ideal camera with 100 µm pixels. The sinc function, defined as $Sinc\left( {rf} \right) = \frac{{Sinc\left( {r\pi f} \right)}}{r\pi f}$, r and f being the pixel pitch and spatial frequency respectively, represents the theoretical upper limit of the MTF by assuming that the pixel aperture is the only image blurring mechanism in the radiography camera. This shows that the imperfections in our detector result in a global average blurring due to light sharing or leakage between five surrounding microcapillaries. The expected Nyquist frequency for 20 µm microcapillaries is 25 lp/mm. To reduce the blurring, we employed microcapillaries with extramural absorption to further improve the spatial resolution and obtain a high MTF at the Nyquist frequency. At 25 l p/mm, we obtained an MTF of 3.2%. Figure S11 includes the MTF plot for the microcapillary-based detector with extramural absorption layers. It shows excellent spatial resolution beyond any known X-ray sensor. However, high X-ray doses were needed to generate these images where the darkening effect from the extramural pattern requires extensive correction, making the use of absorption layers unsuitable for low-dose X-ray imaging.

**Table 1:** Comparison of ultrahigh spatial resolution PEALPB detectors with the current state-of-the-art.

Detector	Detector thickness (µm)	Spatial frequency at 10% MTF	Spatial frequency at 50% MTF
PEALPB (This study)	1200	8.8	5.5
Microcolumnar CsI	200	6	2.1
Pixelated Microcolumnar CsI	700	4.6	1.5
Microcolumnar CsCu_2_I_3_	100	8.7	5.4
Timepix with Si	300	8.6	5.1
a-Se	250	13	6.8
MAPbI_3_	230	10	4.1

The PEALPB detector demonstrates the best thickness to spatial resolution balance, thereby providing an unparalleled solution for detector efficiency and spatial resolution^16–20^.

Further optimization of the camera and coupling of detector plates with pixelated CMOS or a–Si:H backplanes holds the potential to improve the spatial resolution further significantly. We expect these optimized microcapillary-based detectors to perform even better than the results shown in this paper. The combination of efficiency with higher thicknesses and spatial resolution will provide unique solutions to many untapped imaging applications. In addition, these optimized detectors will also be a good candidate for neutron radiography, thereby providing a detector solution for X-ray and neutron multimodal radiography. In principle, any crystalline scintillator can be used instead of PEALPB to fabricate such microcapillary-based, high spatial resolution novel detectors. The next phase of our study will demonstrate such approaches.

## Conclusions

In this study, we have demonstrated the feasibility of high spatial resolution X-ray radiography using PEALPB scintillator-based detectors. Simultaneously achieving high detection efficiency with thick sensors and high spatial resolution is a challenging task for X-ray radiography. The detector configuration comprising thick crystalline PEALPB scintillators in microcapillary plates with pore sizes as low as 20 µm fulfills these criteria and provides an excellent solution for high spatial resolution high-energy X-ray radiography with high frame rates. Potentially, it can also be used for X-ray neutron multimodal radiography. The spatial resolution of the 1200 µm thick PEALPB detectors was determined to be ~ 32% better than 200 µm thick microcolumnar CsI detectors. The spatial resolution was comparable to direct detection devices, such as 300 µm-thick silicon, which are limited in detection efficiency and scalability for large-area radiography applications. Further development and optimization of these structured scintillators will produce ultrahigh spatial resolution detectors with applications in numerous fields.

## Methods

### X-ray imaging

For the characterization of the X-ray performance of the detectors, we used the Thermo Scientific PXS5-928 digitally controlled 90 kV microfocus X-ray source. Reference 1 shows the X-ray setup used for the imaging experiments. Different Al plates were used to achieve the required HVLs for different experiments. A low dose of ~ 1 nGy/s was used for the imaging tests. The various images had acquisition times of between 1 and 30 s. The properties of the CMOS chip used for the imaging experiments are given in Table S2. We have optimized the X-ray imaging using various types of PEALPB detectors, including thin films, thick films and fabric soaked with PEALPB. These X-ray images are shown in Figs. S7–S9. Figure S10 shows the X-ray radiograph taken using the microcapillary-based detector of a 200 µm spring along with the contrast and SNR data in Table S3.

## Supplementary Information

Supplementary Information.

## Data Availability

The datasets generated during and/or analyzed during the current study are available from the corresponding author on reasonable request.
