# Supplementary material for: 2D perovskite-based high spatial resolution X-ray detectors
Source: Sci Rep. 2021 Nov 24;11:22897. doi: 10.1038/s41598-021-02378-w (PMC8613224; doi:10.1038/s41598-021-02378-w)
Supplement: Supplementary file 1 — Supplementary Information. [file 41598_2021_2378_MOESM1_ESM.pdf]

2D Perovskite-Based High Spatial Resolution X-ray Detectors

Amlan Datta[1], John Fiala[1], Shariar Motakef[1]

[1]*CapeSym, Inc., 6 Huron Drive, Natick, MA 01760*

Supplementary Information

S1. Thermal and Fast neutron detection using PEALPB

In addition to X-rays, ^{6}Li-alloyed Phenethylammonium lead bromide (PEALPB) is also responsive to neutrons. The thermal neutrons interact with the ^{6}Li in PEALPB, and an alpha and triton are produced from each interaction. These charged particles then ionize the scintillator resulting in the generation of photons which, like those generated by X-rays, undergo near-complete internal reflection and leave the detector. For fast neutrons with energy E_n primarily undergo elastic scattering with the hydrogen in the PEAPLB and produce recoil protons with energy, $E_p = E_n \cos^2\theta$, θ being the scattering angle. These protons ionize the scintillator and produce the photons that are then detected by the photodetectors.

GEANT4 simulation results

The fast neutron detection efficiency of the microcapillary detector was evaluated by Geant4 Monte Carlo simulations. The detector design architecture is shown in Figure S1. Figure S2 shows the calculated number of photons collected by the imager after firing fast neutrons with an energy of 2.5 MeV, to represent a Deuteron-Deuteron (DD) neutron source, and 14.1 MeV, to represent a DT neutron source, at a series of PEALPB detectors with varying thicknesses practical for radiography. As the thickness of the film increases, more fast neutrons are captured resulting in an increase in the number of photons captured by the imager. The performance of these devices was compared to the performance of stilbene devices of the same thickness (red), and it was observed that PEALPB devices, especially at lower thicknesses, are competitive with stilbene for fast neutron detection.

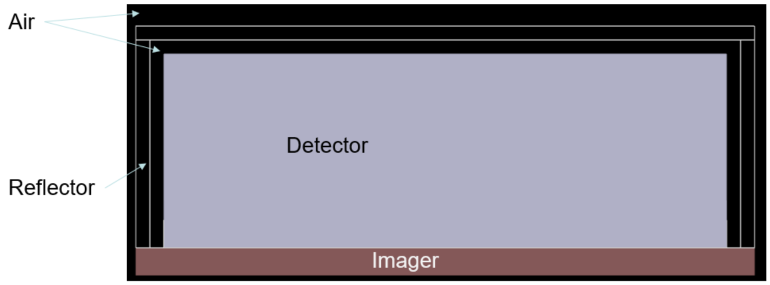

Figure S1. GEANT4 device architecture for fast neutron responses from PEALPB and Stilbene.

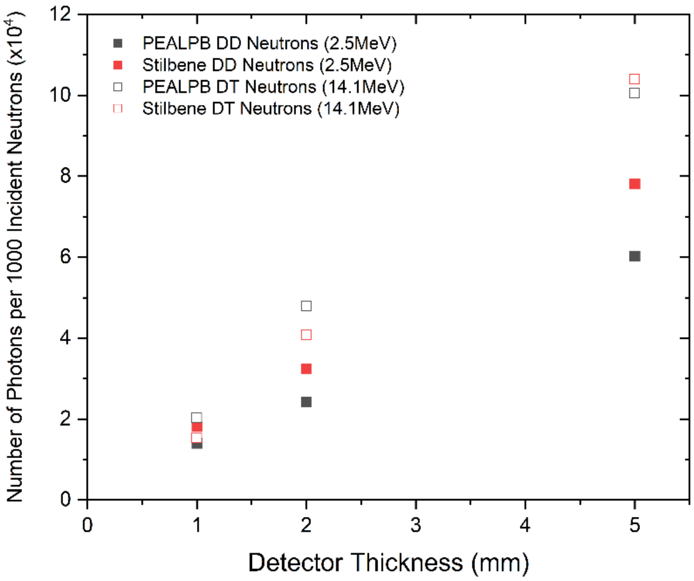

Figure S2. Geant4 results comparing the DD and DT fast neutron responses of PEALPB devices to Stilbene for thicknesses ranging from 1 to 5 mm.

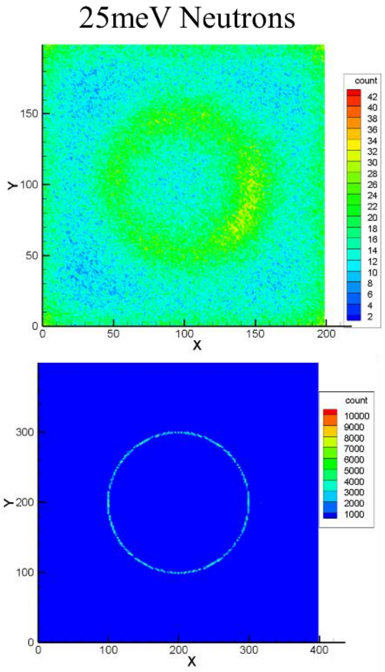

Figure S3. Hit counts of optical photons on pixels of the silicon imager generated using circular pattern of incident 10,000 thermal neutrons (25meV) on the array of PEALPB detectors. Top row hit map shows the light generation without a microcapillary plate and the bottom row shows the same with the microcapillary plate.

DD neutron detection performance

The PEALPB devices fabricated with ^{6}Li were characterized using Neutron irradiation from a Deuterium-Deuterium source. We compared the DD thermal neutron response of the PEAPLB devices with commercial Eljen EJ-410 and EJ-420 fast and thermal neutron detector respectively [1,2]. The neutron characterization set up is shown in Figure S4. All these tests were performed inside a lead cage made with 2-inch thick lead bricks to eradicate any response generated by the X-rays from the DD source and any stray gamma rays. Additional HDPE, Cu sheet, W sheet and Pb sheet shielding is present outside the area shown in this figure. The PMT with the scintillator mated to the top is covered with a light tight packaging to stop any stray light from entering the PMT. DD neutron sources are being used for all the experiments in this paper because these are the industry standard for portable neutron sources used for neutron radiography.

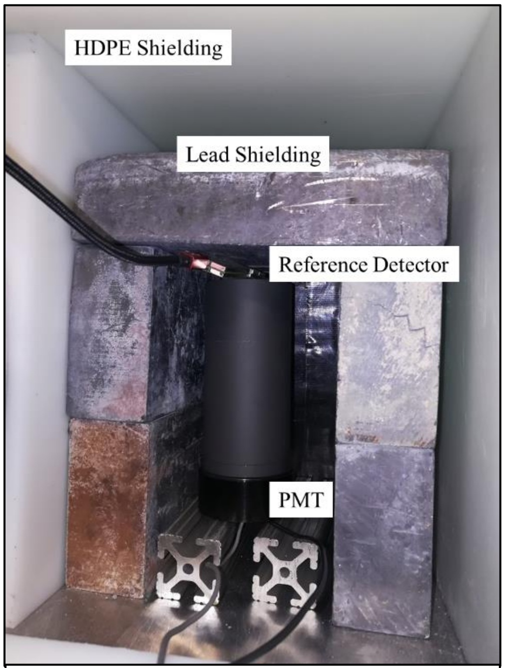

Figure S4. Neutron Detection set up at CapeSym. Additional HDPE, Cu, and W shielding is present outside of this cage and are not shown in this figure.

The thermal neutron response of 1-mm thick PEALPB is compared with that of a 6.5mm-thick Eljen EJ-420 neutron only detector in Figure S5. EJ-420 is fabricated using a ^{6}Li compound dispersed in a ZnS:Ag phosphor matrix. PEALPB has a higher count in the lower energy channels (i.e., for thermal neutrons), and the overall neutron sensitivity as measured by cps/nv is about 30% higher than the thicker EJ-420 detector. For the fast neutron measurements, PEAPB was used without the lithium doping to eliminate thermal neutron detection probability from the ^{6}Li interactions. Figure S6 shows the response of 1-mm thick PEALPB to fast neutrons and compares it with the response of 16-mm thick EJ-410 fast neutron detector with similar cross-sectional area. At higher channels, the histogram counts are similar for both the scintillators but at lower channels, the aggregate counts of PEAPB is 1.5 times higher than EJ-410. From this data, it is clear that the PEALPB detectors can easily replace the currently used ZnS phosphor-based neutron radiography screens. With the in-house DD source, we could not acquire neutron images using PEALPB or $^{6}LiF/ZnS$

detector plates similar to the X-ray images as discussed in the latter part of this section. This is because the neutron source did not have a high enough flux and directionality required for neutron imaging.

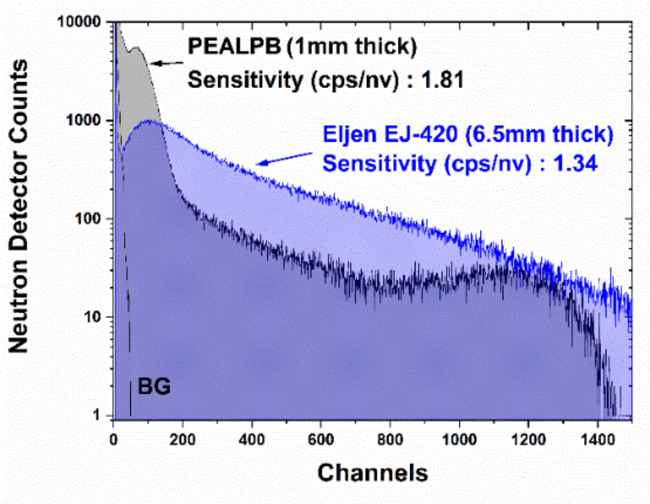

Figure S5. Thermal neutron response of 1mm thick PEALPB and its comparison with commercial EJ-420 thermal neutron detectors.

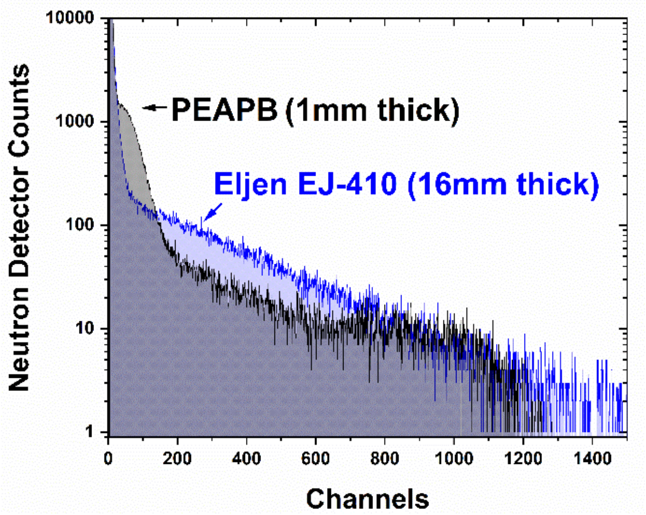

Figure S6. Fast neutron response of 1mm thick PEAPB and its comparison with commercial EJ-410 fast neutron detectors.

S2. PEALPB X-ray detector results

Table S1. Uniformity data for the seven ROIs measured from the background image.

ROI	Mean	Standard Deviation
1	157.19	47.82
2	156.86	47.92
3	157.76	48.98
4	156.02	46.47
5	156.24	47.81
6	155.43	46.93
7	157.45	48.58

Table S2. Properties of the CMOS chip used for fabricating the X-ray imaging camera.

Number of Pixels	4096 x 3000
Optical Format	17.5 mm
Pixel Size	3.45µm x 3.45µm
Full Well Capacity	>10,650 e^-
Dynamic Range	71dB

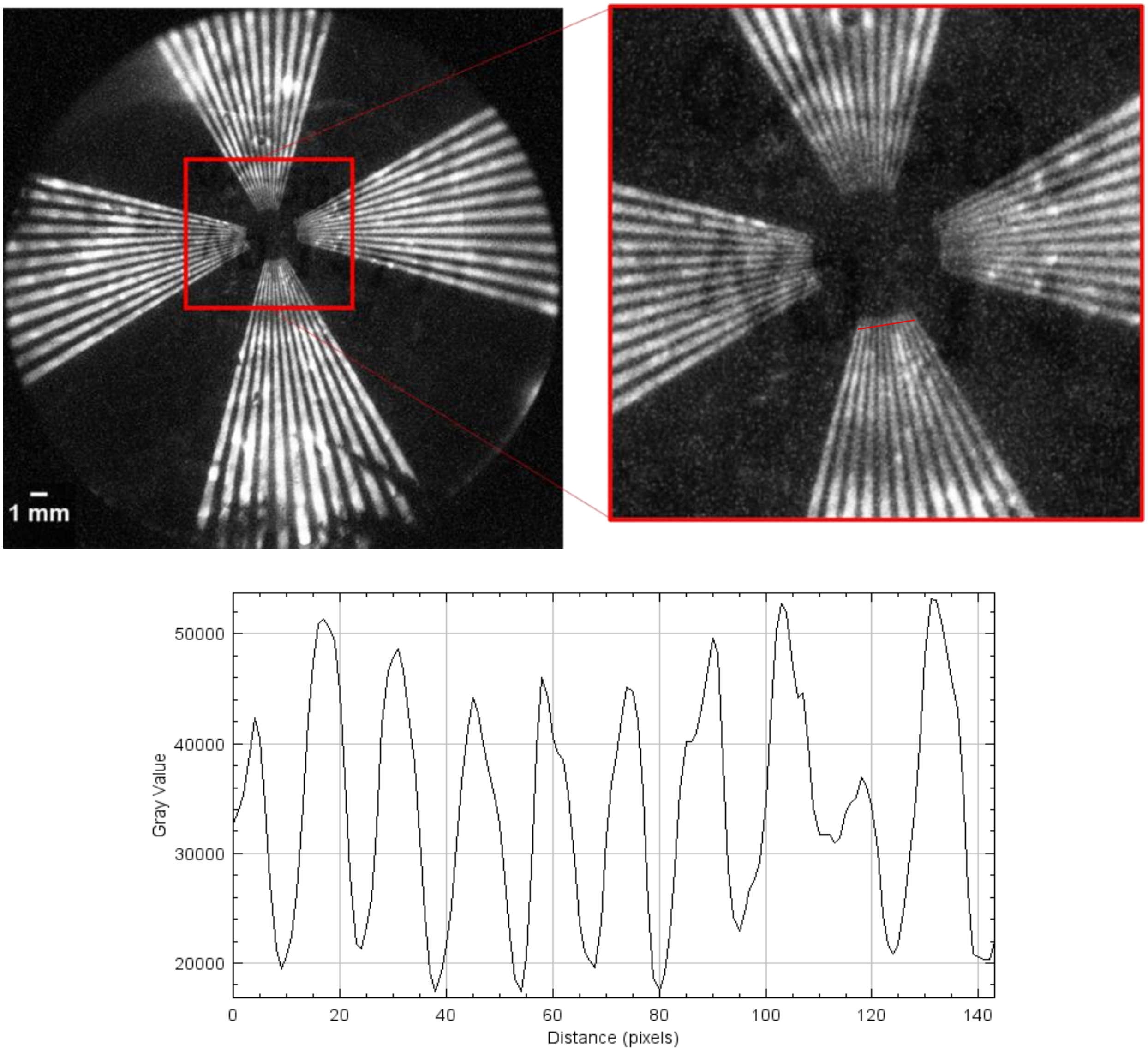

Figure S7. (Top) Star pattern X-ray image of the Siemens star target showing excellent spatial resolution of PEALPB thin films. The innermost lines have a Nyquist frequency of 10lp/mm. (Bottom) The line profile at 10lp/mm frequency as collected from the red line of the magnified image.

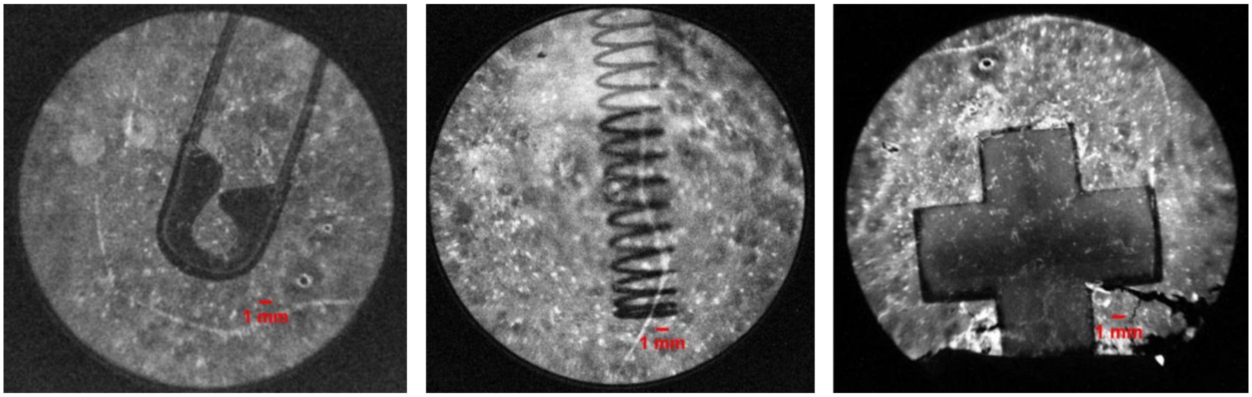

Figure S8. X-ray radiographs of a safety pin, spring, and magnetic stirrer were acquired using PEALPB thick film.

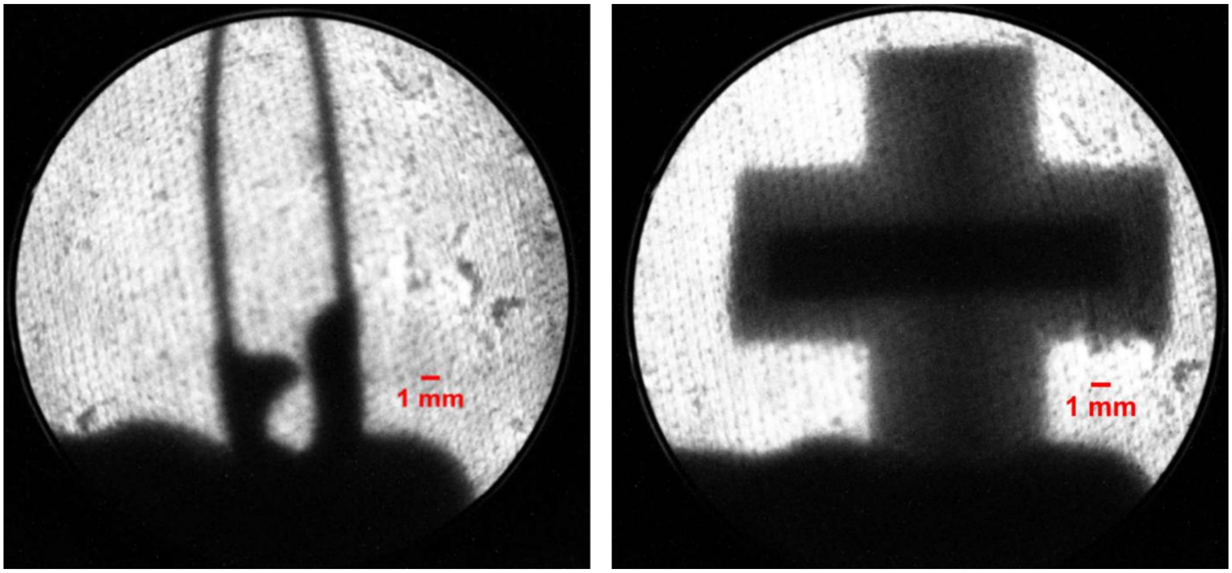

Figure S9. X-ray radiographs of a safety pin and a magnetic stirrer were acquired using a PEALPB infused fabric.

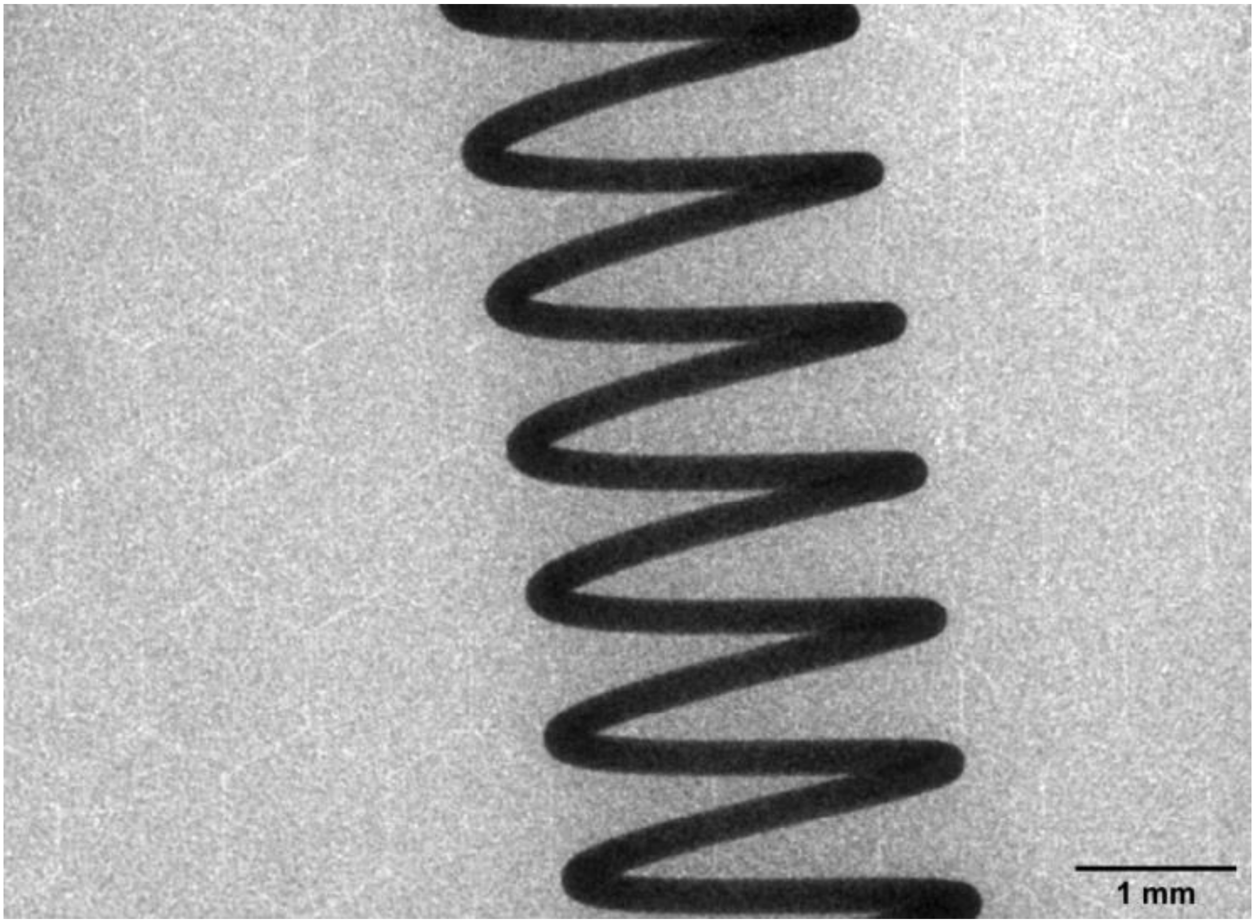

Figure S10. X-ray image of a 200µm-thick spring using the PEALPB camera. The following table S3 gives the SNR and relative contrast values for different areas of this image.

Table S3. SNR and relative contrast values of the 200µm-thick spring radiograph.

ROI	Mean SNR	Contrast
1	123	6.17
2	152	6.22
3	109	5.92
4	104	5.88

S3. Performance of Microcapillary based Sensors with Extramural Absorption

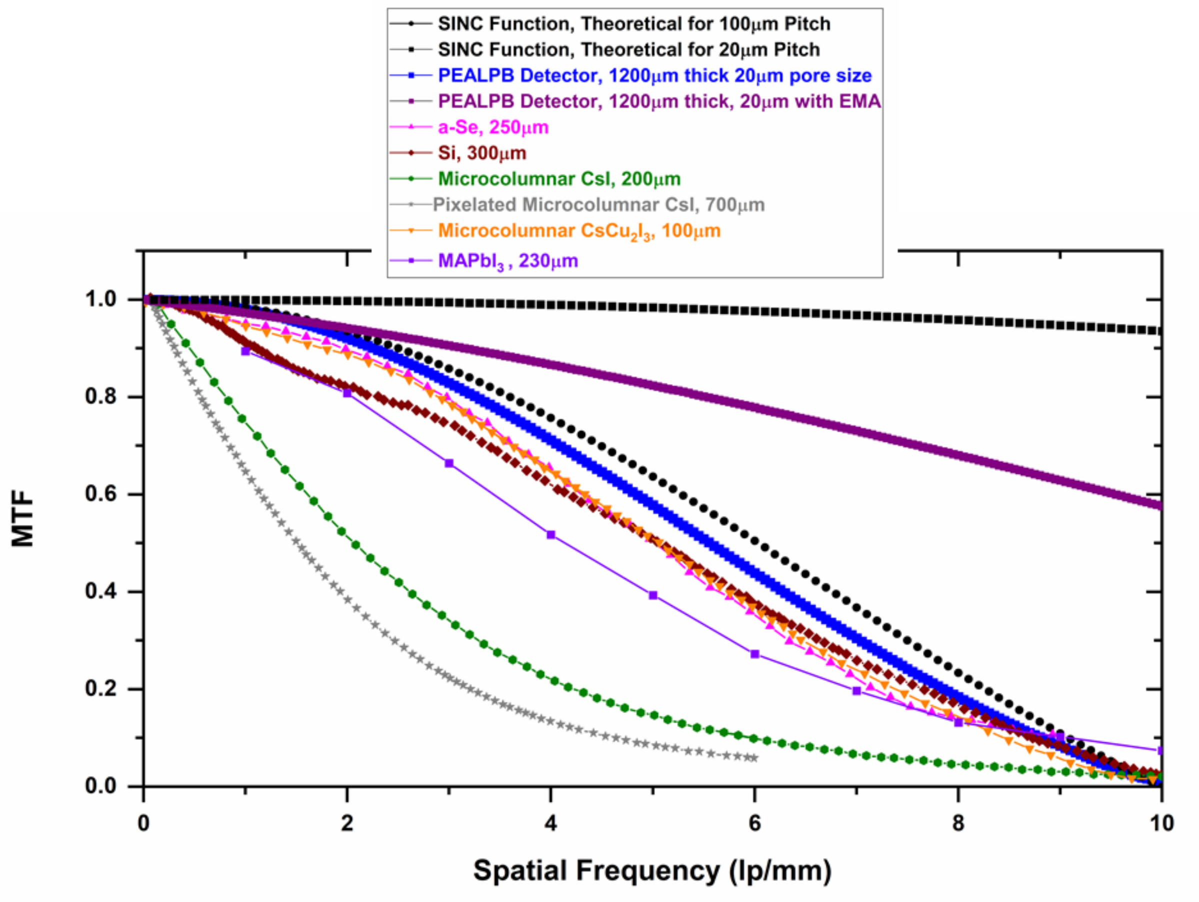

Figure S11. MTF curve of PEALPB detectors with and without extramural absorption layers. This approach provides excellent spatial resolution, however needs high X-ray dose and the images need extensive correction to eliminate the shadows generated by the extramural layers.

S4. References:

[1] https://eljentechnology.com/products/neutron-detectors/ej-420

[2] https://eljentechnology.com/products/neutron-detectors/ej-410
